# White Matter Development in Early Puberty: A Longitudinal Volumetric and Diffusion Tensor Imaging Twin Study

**DOI:** 10.1371/journal.pone.0032316

**Published:** 2012-04-13

**Authors:** Rachel M. Brouwer, René C. W. Mandl, Hugo G. Schnack, Inge L. C. van Soelen, G. Caroline van Baal, Jiska S. Peper, René S. Kahn, Dorret I. Boomsma, H. E. Hulshoff Pol

**Affiliations:** 1 Department of Psychiatry, Rudolf Magnus Institute of Neuroscience, University Medical Center Utrecht, Utrecht, The Netherlands; 2 Department of Biological Psychology, VU University Amsterdam, Amsterdam, The Netherlands; 3 Brain and Development Laboratory, Leiden University, Leiden, The Netherlands; Beijing Normal University, Beijing, China

## Abstract

White matter microstructure and volume show synchronous developmental patterns in children. White matter volume increases considerably during development. Fractional anisotropy, a measure for white matter microstructural directionality, also increases with age. Development of white matter volume and development of white matter microstructure seem to go hand in hand. The extent to which the same or different genetic and/or environmental factors drive these two aspects of white matter maturation is currently unknown. We mapped changes in white matter volume, surface area and diffusion parameters in mono- and dizygotic twins who were scanned at age 9 (203 individuals) and again at age 12 (126 individuals). Over the three-year interval, white matter volume (+6.0%) and surface area (+1.7%) increased, fiber bundles expanded (most pronounced in the left arcuate fasciculus and splenium), and fractional anisotropy increased (+3.0%). Genes influenced white matter volume (heritability ∼85%), surface area (∼85%), and fractional anisotropy (locally 7% to 50%) at both ages. Finally, volumetric white matter growth was negatively correlated with fractional anisotropy increase (r = –0.62) and this relationship was driven by environmental factors. In children who showed the most pronounced white matter growth, fractional anisotropy increased the least and vice-versa. Thus, white matter development in childhood may reflect a process of both expansion and fiber optimization.

## Introduction

During development, the composition of the brain changes substantially. After a remarkable increase in white matter volume in the first few years of life, both gray and white matter of the brain have been found to steadily increase during childhood, using conventional anatomical magnetic resonance imaging. While gray matter volume starts to decrease around the start of puberty, white matter volume continues to increase well into adulthood [1]–[3]. Indeed, at the age when thinning of the cortex has already started [4]–[5], white matter expands considerably. The biological processes that underlie these white matter changes are still largely unknown.

With diffusion tensor imaging (DTI;[6]) the white matter of the brain can be studied in more detail compared to conventional magnetic resonance brain imaging. DTI quantifies diffusion of water molecules in the brain. In white matter bundles, water diffuses more easily in the direction parallel to axons (axial diffusivity) than in the direction perpendicular to axons (radial diffusivity). Fractional anisotropy [7] is a combination of these two and represents microstructural directionality, or so-called ‘fiber integrity’. White matter microstructural development, as measured with DTI, shows a pattern parallel to the development of white matter volume. Numerous studies have been carried out to investigate age-related changes in diffusion properties in childhood and adolescence. Despite differences in methodology and diffusion parameters that were studied, these studies all indicate that anisotropy increases with age during development, with the fastest increases in infancy and young childhood ([8]–[9]; see [10]–[12] for reviews on earlier studies). These cross-sectional findings have been replicated in three recent longitudinal studies ([13] age 16–21; [14] age 14–19; [15] age 5–32). To summarize, the development of white matter volume and of white matter microstructure follow the same pattern and seem to go hand in hand.

Small to moderate associations between white matter volume and diffusion in the brain in adolescents were found in a single study that employed a cross-sectional design [16]. So far, the etiology of these associations has not been studied. In our study, we explored the development of white matter in children using a longitudinal design with a 3-year test-retest interval. We studied local changes in white matter volume and in white matter microstructure in a sample of twins between the age of 9 and 12 years using magnetic resonance brain imaging. The following questions were addressed: 1) How do white matter volume and white matter microstructure change between the ages of 9 and 12? Are these quantities and/or their changes related? 2) What is the heritability of white matter volume, white matter microstructure and their changes? If these are associated, are these relationships genetic or environmental in nature?

## Materials and Methods

### Subjects

Participating in this study were 108 twin families. Twins were first recruited at 9 years of age through the Netherlands Twin Registry [17]. At the first measurement at age 9 (mean 9.2, sd 0.11 years), 203 twin subjects (88 monozygotic twin subjects, 43 complete pairs and 115 dizygotic twin subjects, 53 complete pairs - 101 males, 102 females) underwent an extensive MRI protocol, as was described before [18]–[19]. Exclusion criteria consisted of having a pacemaker, any metal material in the head and a known history of any psychiatric illness or major medical condition. Psychiatric and medical status was confirmed through a medical questionnaire filled in by the parents at the time of MRI scanning. Three years later, 126 twin subjects returned at age 12 (mean 12.1, sd 0.24 years) (59 monozygotic twins, 25 complete pairs and 67 dizygotic twin subjects, 27 complete pairs - 64 males, 62 females). Mean scanning interval was 2.9 (0.2) years. Zygosity of same-sex twins was determined based on DNA polymorphisms, using 8–11 highly polymorphic di-, tri- and tetranucloide genetic markers. Handedness was determined according to the Edinburgh Handedness Inventory. At baseline 84% of the sample was right-handed; at follow-up 83%. Both parents and children gave written informed consent to participate in the study. The study was approved by the Central Committee on Research involving Human Subjects of the Netherlands (CCMO) and was in agreement with the Declaration of Helsinki (Edinburgh amendments).

### MRI Acquisition and Preprocessing

Magnetic resonance imaging and post-processing of the data was done at the University Medical Center, Utrecht. For both measurements, structural magnetic resonance images were made on a 1.5 Tesla Philips Achieva scanner (Philips, Best, the Netherlands) using the same protocol. A three-dimensional T1-weighted scan (Spoiled Gradient Echo; TE = 4.6 ms; TR = 30 ms; flip angle 30°; 160–180 contiguous coronal slices of 1.2 mm; in-plane resolution 1×1 mm^2^; acquisition matrix 256×256) of the whole head was made of each subject. To increase signal to noise ratio, two Single Shot Echo Planar Imaging (SS-EPI) DTI scans were acquired (32 diffusion-weighted volumes with diffusion weighting b = 1000 s/mm^2^ and 32 non-collinear diffusion gradient directions; 8 diffusion-unweighted (b = 0 s/mm^2^) scans; TE = 88 ms; TR = 9822 ms; parallel imaging SENSE factor 2.5; flip angle 90°; 60 transverse slices of 2.5 mm, no gap, FOV 240 mm; 128×128 reconstruction matrix; 96×96 acquisition matrix, no cardiac gating). The DTI scans were combined and corrected for geometric distortions [20], but due to the use of parallel imaging with a short echo time at 1.5 Tesla, susceptibility distortions are expected to be minimal. Subsequently, the diffusion pattern in each voxel was fitted to a tensor matrix using a robust M-estimator [21], providing three eigenvectors (representing the three principal directions of diffusion) and corresponding eigenvalues (λ_1_>λ_2_≈λ_3_, in white matter). Fractional anisotropy values were calculated in each voxel as a measure of microstructural directionality from the eigenvalues [7]. Further, axial/longitudinal (λ_1_) and radial/transverse ((λ_2_+λ_3_)/2) diffusivity were computed.

To obtain white matter volume and mean white matter fractional anisotropy, gray and white matter were segmented from the T1-weighted images using a partial volume segmentation [22]. Reliable gray and white matter segmentations were available in 187 children at baseline, and 117 children at follow-up. A pure white matter mask was created by selecting all voxels that were classified as having at least 90% white matter content. For computation of the mean fractional anisotropy in pure white matter, the b0 scan was registered to the T1-weighted scan using a rigid transformation (no scaling), based on optimization of a mutual information metric [23]. Possible local DTI distortions therefore may be expected to have limited influence on the registration. Diffusion parameters were warped on the T1-weighted scan using this rigid transformation and mean fractional anisotropy in pure white matter was computed by averaging over the voxels in the pure white matter mask.

### Deformation Based Morphometry

To study morphological changes between baseline and follow-up, the deformation fields of the T1-weighted images from baseline to follow-up were computed using a combination of linear and non-linear transformations [24] with increasing precision up to the scanning resolution. The determinants of the Jacobian matrix *J* of this these deformation fields were computed, which were used to assess expansion or contraction of tissue. The determinant maps were warped into model space, again with a precision up to scanning resolution. The model brain was created using the T1-weighted images from the follow-up measurement, (analogous to [25]). Voxels were resampled to a resolution of 2×2×2.4 mm^3^. Each voxel was tested for volumetric change (either expansion or contraction, |*J*|≠1). A correction for multiple comparisons was applied according to the false discovery rate (FDR; [26]) at a 0.05 level.

### Fiber Tractography

Analogous to the analysis at the baseline measurement [19] we created average fiber tracts for the whole group containing scans for both measurements, to study parts of the fiber tract that are common to most subjects [27]–[28]. First, all possible fiber tracts in the brain were reconstructed in individual space using the FACT algorithm (Fiber Assignment by Continuous Tracking; [29]), with 8 seedpoints per voxel (taking the corners of small cubes inside a voxel as seed points, placed such that the smaller cubes form a regular grid), a fractional anisotropy threshold of 0.1 and maximal angle of 45°. These settings were chosen to perform rather liberal fiber tracking. Subsequent fiber averaging removes superfluous tracts. Per subject, all reconstructed tracts were superimposed with axial diffusivity, radial diffusivity and fractional anisotropy values. Next, for each subject, the complete set of tracts – which was reconstructed in native space – was warped into model space using a nonlinear transformation. For each subject this nonlinear transformation was obtained by concatenating the (linear) transformation that registers the b0 image to the T1-weighted image (in native space) with the (nonlinear) transformation that warps the T1-weighted image to the model brain. Finally, multiple regions of interest (ROIs) were defined in model space allowing us to select 14 different major fiber tracts for each subject: the arcuate fasciculi, uncinate fasciculi, fornices, cinguli, inferior longitudinal fasciculi and inferior fronto-occipital fasciculi bilaterally, and the genu and splenium of the corpus callosum [28] [30].

### Creation of Average Fibers

For each individual separately, we created an average fiber bundle at group level for the 14 major fiber tracts as described in [28]. In short, for each fiber bundle, the middle points of all fibers in the bundle were determined and the (spatial) average served as a starting point for the average fiber. Subsequently, the n^th^ coordinate of the average fiber was computed as the spatial average of the points in the fiber bundle at distance 2*n* mm from the starting point. The individual average fibers were smoothed and resampled to their original resolution. As tracking results may vary per individual, the individual average fibers bundles (combining all children at both measurements) were averaged again. Because the outer ends of fiber bundles may differ per individual close to gray matter, we ignored parts of this average fiber to which less than 25% of the sample contributed. This procedure created a (spatial) average fiber bundle at group level. Now, for each individual and each fiber bundle, the fiber bundles were projected onto their respective group averages. If the individual fiber tracking results were too far (>1 cm) from the average fiber, we considered this measurement to be unreliable. Therefore, not all fiber bundles could be traced in all children (see Tables 2–4 for exact numbers). Mean fractional anisotropy, axial diffusivity and radial diffusivity along these bundles was obtained per subject. For the comparison of white matter volumetric measures and DTI quantities, masks of fiber bundles were created in model space as follows: For each individual, voxels in model space were flagged according to whether the individual average fiber bundle passed through these voxels. These individual masks were summed and were subsequently used to create weighted averages per individual of the Jacobian in this fiber bundle.

### Surface Area Measures

White matter surface area was computed using a custom version of the CLASP algorithm, designed at the McConnell Brain Imaging Centre, Montreal [31]–[32], which started from the gray and white matter segments created by our own algorithm as described above. A 3D surface consisting of 40962 vertices was fitted to the white matter/gray matter interface, which created the inner surface of the cortex, i.e. the white matter surface [31]. Region-of-Interests (ROIs) were automatically segmented using the automated anatomical labelling (AAL) atlas [33] resulting in 78 ROIs (39 for both left and right hemispheres). Total, lobular and regional white matter surface area was computed for each individual.

### Statistical analysis

Changes over time were evaluated using paired t-tests. Absolute and relative change measures over time were computed for white matter volume, white matter surface area and mean fractional anisotropy in pure white matter. Pearson’s correlations were used when studying the associations between these change measures. In all non-genetic analyses, the degrees of freedom were adjusted to account for familial dependencies in the data, using the number of families instead of the number of children as the sample size.

### Twin Data - Structural Equation Modeling

Twin data can be used to estimate the respective contributions of genetic and unique environmental influences on a certain trait [34], as well as on the covariance between traits [35]. Genetic influence cause biological relatives to resemble each other, as do environmental influences that are shared by family members. Environmental factors that are not shared cause differences among family members. As monozygotic (MZ) twins share (almost) 100% of their segregating genes and dizygotic (DZ) twins share on average 50% of their segregating genes, a higher correlation between members of MZ twin pairs than between members of DZ twin pairs indicates that genes, rather than shared environment, play a role in explaining the resemblance between co-twins. The proportion of variance that can be attributed to genetic factors is called *heritability*. Amongst the assumptions of the twin design are the equal environment assumption (MZ twin pairs are not treated differently by their environment than DZ pairs) and random mating (in this case, parents did not choose their mates based on brain size or fractional anisotropy) [34].

Heritability estimates for white matter volume, white matter surface area and mean fractional anisotropy in pure white matter were obtained by structural equation modeling of data obtained at age 9 and age 12 years. A bivariate genetic model specified traits at both ages to be influences by additive genetic factors and by unique environment (for a detailed description of such a model, see e.g [36]). Heritability estimates of fractional anisotropy in fiber bundles were obtained through a (multivariate) Cholesky decomposition [37] analogous to the analysis at baseline [19]. To limit the amount of variables in one analysis, we performed three separate analyses: one for callosal fibers (genu and splenium of corpus callosum), one for fibers in the left hemisphere (left arcuate, cingulum, fornix, IFO, ILF, and uncinate) and one for fibers in the right hemisphere (right arcuate, cingulum, fornix, IFO, ILF, and uncinate). All three analyses included both baseline and follow-up measures. Confidence intervals for heritability estimates were obtained through maximum likelihood estimation, with lower and upper bound being the point for which the log-likelihood changes more than 2.71.

Next, observed correlations between change measures over time were investigated to determine whether they are genetic or environmental in origin: larger cross-twin cross-trait correlations in MZ twins compared to DZ twins indicate that the association is driven by genetic sources. Genetic (r_g_) and environmental (r_e_) correlations, representing the amount of overlap between genetic and environmental factors that influence both traits, were derived from the bivariate model [25]. All genetic analyses were performed in Mx [38]. All genetic analyses included sex and handedness as fixed effects on the means.

### Post-hoc Analysis: Histogram Comparisons

It can be suggested that the inverse relationship found between white matter growth and fractional anisotropy increase may be the results of segmentation: it could be the case that in children that showed to most pronounced volumetric white matter growth, more regions of crossing fibers are included in the white matter mask. This would results in a lower mean fractional anisotropy. To test this, we selected two groups of children with “extreme” volumetric white matter changes: one consisting of children with a relatively large volumetric growth (>mean+1 s.d.) (13 children) and one of children with a relatively small volumetric white matter growth (<mean–1 s.d.) (14 children). Instead of selecting white matter in individual space, we warped all images to the model brain, on which we selected pure white matter. Fractional anisotropy was therefore measured in the same areas and differences cannot originate from the individual segmentations. Then we computed FA histograms in the pure white matter mask. If the above were true, an increase in low FA values should be observed in the group of children with relatively large white matter growth.

## Results

There were no differences in sex, handedness, age at baseline/follow-up or zygosity between children that were measured twice and children who participated only once (p’s>0.17). See Table 1 for demographics.

**Table 1:** Sample characteristics of the full sample (left) and of the subset of children for which longitudinal data was available (right).

	Full sample		Longitudinal sample		p-value
	Age 9	Age 12	Age 9	Age 12	
Number of twin subjects	203	126	121	121	
(MZ / DZ)	(88/115)	(59/87)	(57/64)	(57/64)	0.20
Mean age	9.2	12.1	9.2	12.1	0.17 (age 9)
(sd) in years	(0.11)	(0.24)	(0.11)	(0.25)	0.68 (age 12)
Sex (boy/girl)	101/102	64/62	63/58	63/58	0.33
Handedness (R/non-R)	171/32	105/21	101/20	101/20	0.85
Mean FA in pure WM	0.401	0.413	0.401	0.414	0.59 (age 9)
(sd) */**	(0.02)	(0.02)	(0.02)	(0.02)	0.69 (age 12)
Mean WM volume	512.9	543.8	517.3	544.3	0.24 (age 9)
(sd) in ml */**	(57.9)	(63.3)	(59.7)	(62.3)	0.83 (age 12)
Mean WM surface area	1908.6	1940.1	1913.5	1939.6	0.67 (age 9)
(sd) in cm^2^ */**	(177.7)	(179.4)	(182.8)	(180.5)	0.94 (age 12)

MZ  =  monozygotic, DZ  =  dizygotic, FA  =  fractional anisotropy, R = right-handed, WM  =  white matter.

* White matter was segmented reliably in 187 children at baseline, and 117 children at follow-up.

** 105 children had reliable white matter segmentations and DTI measurements at both time-points. The last column displays p-values of the differences in sample characteristics for children who participated only once, versus children who participated twice.

### Volumetric White Matter Growth

**Figure 1:**
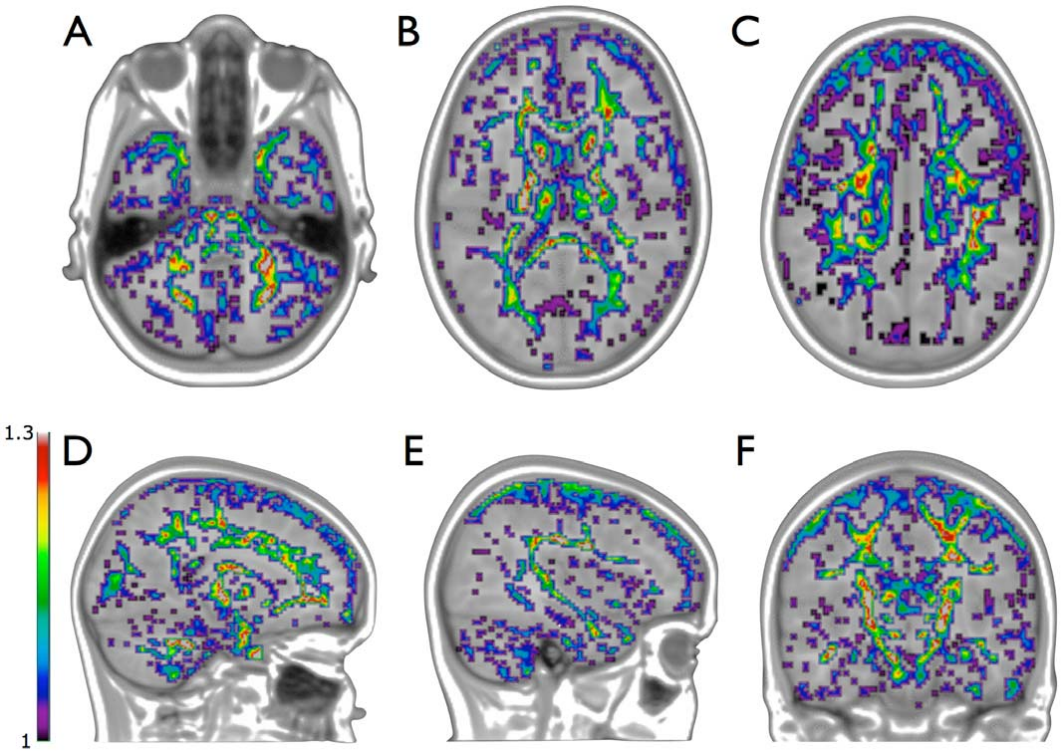
Significant tissue expansion: Significant Jacobian values > 1 overlaid on the model brain. Values range from 1.02 (purple) to 1.30 (bright red). Expansion was observed in regions covering the arcuate fasciculus (E), cerebellar penduncle (A), cinguli (C) , corpus callosum (B,C), cortical spinal tract (B,F), uncinate fasciculus (D) and on the gray matter / cerebral spinal fluid boundary (A-F), the latter likely representing brain growth. For visualization purposes, values were resampled to model brain resolution.

White matter volume of the total brain increased on average from 512.9 ml (s.d. 57.9) to 543.9 ml (s.d. 63.3) (6.0%, p<0.001) between baseline and follow-up. Locally, the deformation-based analysis revealed large significant clusters of white matter tissue expansion (|*J|*>1) in which many major fiber bundles are visible (|t|>2.37, FDR corrected). These include areas covering the corpus callosum, the cortical spinal tracts, the cerebellar peduncle, bilateral cinguli, bilateral superior longitudinal fasciculi (including the arcuate fasciculi), bilateral fornices, bilateral inferior longitudinal fasciculi/posterior thalamic radiation, bilateral inferior fronto-occiptal fasciculi, and bilateral uncinate fasciculi (Figure 1). Tissue contraction occurs in gray matter at the gray/white matter interface and around the ventricles, see Figure S1.

Averaging the Jacobians over the fiber bundles, lengthening and thickening of the bundles was found (|*J|*>1, p’s<1×10^–6^), with the most pronounced expansion in the left arcuate fasciculus (|*J|* = 1.08), and the splenium of the corpus callosum (|*J|* = 1.08) and the least prominent expansion in the left and right cinguli (|*J|* = 1.02 for both).

### Microstructural White Matter Changes

**Figure 2:**
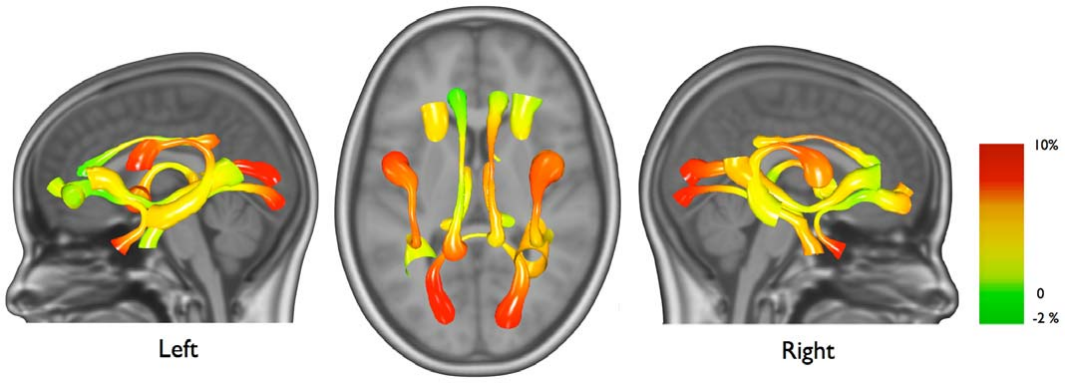
Increases of relative fractional anisotropy between the ages of 9 and 12, projected onto the group average fiber bundle. Left, top and right view. For visualization purposes, values were smoothed along the bundle using LOESS [59].

In all 14 fiber tracts, fractional anisotropy increased (on average between 1.3% and 6.9%, Table 2; Figure 2) (p’s<0.012). In most fibers, the increase in fractional anisotropy is due to both an increase in axial diffusivity and a decrease in radial diffusivity (Tables 3 and 4). A notable exception is the genu of the corpus callosum where the increase in fractional anisotropy seems mainly driven by an increase in axial diffusivity. Following the pattern observed in individual fiber bundles, mean fractional anisotropy in pure white matter increased from 0.40 to 0.41 (3.0%, p<0.001). This increase can be attributed to a decrease in radial diffusivity.

**Table 2:** Fractional anisotropy in 9 and 12 year old children.

	N	9 years	12 years	p	Change
Pure white matter	105	0.40 (0.03)	0.41 (0.02)	<0.0001	+3.0%
Arcuate L	117	0.48 (0.03)	0.50 (0.03)	<0.0001	+4.6%
Arcuate R	99	0.48 (0.03)	0.50 (0.03)	<0.0001	+5.3%
Cingulum L	83	0.50 (0.06)	0.52 (0.05)	0.0120	+3.2%
Cingulum R	74	0.45 (0.06)	0.48 (0.06)	<0.0001	+6.9%
Fornix L	114	0.34 (0.03)	0.36 (0.03)	<0.0001	+4.9%
Fornix R	111	0.34 (0.03)	0.35 (0.03)	<0.0001	+4.7%
Genu	121	0.61 (0.03)	0.62 (0.03)	0.0052	+1.3%
IFO L	120	0.51 (0.03)	0.52 (0.03)	0.0002	+2.6%
IFO R	121	0.48 (0.03)	0.50 (0.03)	<0.0001	+3.7%
ILF L	121	0.48 (0.03)	0.49 (0.03)	<0.0001	+3.1%
ILF R	121	0.48 (0.03)	0.49 (0.03)	<0.0001	+4.2%
Splenium	121	0.60 (0.04)	0.63 (0.04)	<0.0001	+5.5%
Uncinate L	113	0.45 (0.03)	0.46 (0.03)	<0.0001	+3.1%
Uncinate R	120	0.43 (0.03)	0.45 (0.03)	<0.0001	+3.2%

Means and standard deviations are given from the longitudinal sample only.

L = left, R = right, ILF = inferior longitudinal fasciculus, IFO = inferior fronto-occipital fasciculus. P-values are the result of paired t-tests comparing fractional anisotropy at 9 and 12 years. N represents the number of children included in the paired t-test.

**Table 3 pone-0032316-t003:** Longitudinal diffusivity (λ_1_; x 10^−3^ mm^2^/s) in 9 and 12 year old children.

	N	9 years	12 years	p	Change
Pure white matter	105	1.139 (0.032)	1.143 (0.028)	0.227	+0.4%
Arcuate L	117	1.199 (0.052)	1.199 (0.044)	0.752	–0.1%
Arcuate R	99	1.180 (0.051)	1.193 (0.055)	0.014	+1.2%
Cingulum L	83	1.209 (0.085)	1.245 (0.079)	0.001	+3.0%
Cingulum R	74	1.163 (0.078)	1.210 (0.093)	<0.001	+4.0%
Fornix L	114	1.360 (0.119)	1.372 (0.136)	0.389	+0.9%
Fornix R	111	1.375 (0.195)	1.387 (0.156)	0.575	+0.8%
Genu	121	1.351 (0.063)	1.390 (0.045)	<0.001	+2.8%
IFO L	120	1.251 (0.054)	1.269 (0.046)	0.002	+1.4%
IFO R	121	1.229 (0.055)	1.267 (0.048)	<0.001	+2.2%
ILF L	121	1.314 (0.074)	1.323 (0.061)	0.152	+0.8%
ILF R	121	1.318 (0.068)	1.336 (0.057)	0.002	+1.4%
Splenium	121	1.582 (0.086)	1.612 (0.084)	<0.001	+2.2%
Uncinate L	113	1.185 (0.050)	1.203 (0.039)	0.001	+1.5%
Uncinate R	120	1.177 (0.050)	1.199 (0.044)	<0.001	+1.9%

Means and standard deviations are given from the longitudinal sample only.

L = left, R = right, ILF = inferior longitudinal fasciculus, IFO = inferior fronto-occipital fasciculus. P-values are the result of paired t-tests comparing λ_1_ at 9 and 12 years. N represents the number of children included in the paired t-test.

**Table 4:** Radial diffusivity (λ_23_; ×10^–3^ mm^2^/s) in 9 and 12 year old children.

	N	9 years	12 years	p	Change
Pure white matter	105	0.596 (0.021)	0.584 (0.018)	<0.001	–2.1%
Arcuate L	117	0.542 (0.028)	0.520 (0.027)	<0.001	–4.0%
Arcuate R	99	0.535 (0.028)	0.516 (0.027)	<0.001	–4.0%
Cingulum L	83	0.506 (0.044)	0.503 (0.037)	0.459	–0.6%
Cingulum R	74	0.544 (0.053)	0.533 (0.045)	0.010	–2.6%
Fornix L	114	0.811 (0.088)	0.789 (0.098)	0.030	–2.7%
Fornix R	111	0.830 (0.135)	0.813 (0.105)	0.278	–1.9%
Genu	121	0.433 (0.037)	0.439 (0.030	0.067	–1.3%
IFO L	120	0.531 (0.033)	0.520 (0.028)	0.001	–1.9%
IFO R	121	0.552 (0.034)	0.541 (0.027)	<0.001	–1.9%
ILF L	121	0.591 (0.040)	0.577 (0.045)	0.003	–2.3%
ILF R	121	0.597 (0.045)	0.579 (0.040)	<0.001	–3.0%
Splenium	121	0.547 (0.069)	0.513 (0.061)	<0.001	–6.3%
Uncinate L	113	0.563 (0.031)	0.555 (0.025)	0.002	–1.6%
Uncinate R	120	0.574 (0.030)	0.568 (0.026)	0.008	–1.0%

Means and standard deviations are given from the longitudinal sample only.

L = left, R = right, ILF = inferior longitudinal fasciculus, IFO = inferior fronto-occipital fasciculus. P-values are the result of paired t-tests comparing λ_23_ at 9 and 12 years. N represents the number of children included in the paired t-tests.

### The Relation between White Matter Volume and Fractional Anisotropy

**Figure 3:**
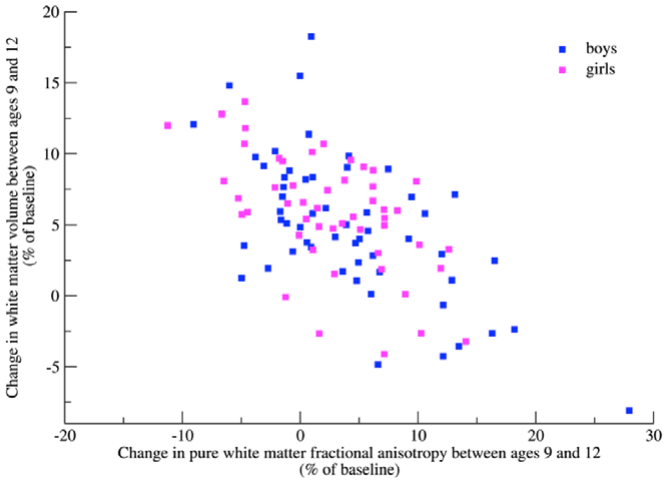
The relationship between *changes* in pure white matter fractional anisotropy and *changes* in white matter volume in the three-year interval.

There was no significant correlation between white matter volume and mean fractional anisotropy in pure white matter at either baseline or follow-up, or between white matter volume and mean pure white matter radial or axial diffusivity (p’s>0.21). However, there was a strong negative correlation between the relative volumetric white matter growth and the relative fractional anisotropy increase (r_p_ = –0.62, p<0.001, see Figure 3). This correlation is a reflection of the associations between the relative volumetric white matter change and relative radial diffusivity change (r_p_ = 0.45, p<0.001) and relative axial diffusivity change (r_p_ = –0.47, p<0.001). Next, we investigated the relation between the baseline measurement and subsequent changes. The baseline measurement of white matter volume predicted the relative increase in fractional anisotropy over time (r_p_ = 0.27, p = 0.013). Similarly, mean fractional anisotropy at age 9 predicted the relative increase in white matter volume.

### White Matter Surface Area Expansion and the Relation to Fractional Anisotropy

**Figure 4:**
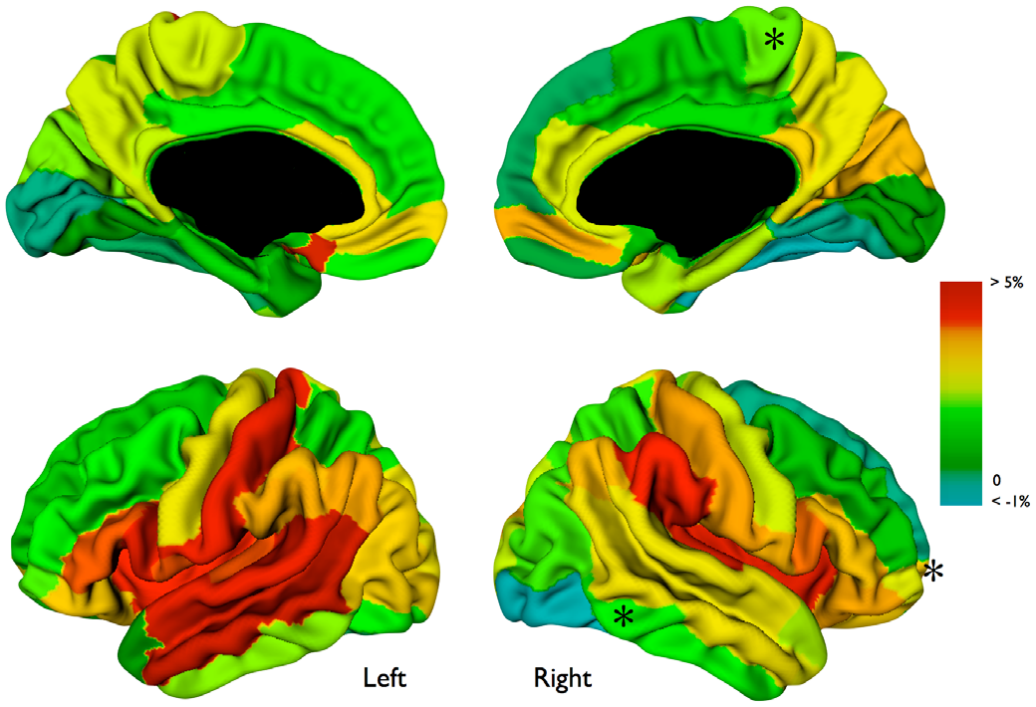
Relative white matter surface area expansion of AAL regions in the left and right hemisphere. Please note that expansion is based (and projected on) AAL regions rather than representing continuous expansion. *Areas in which girls had larger expansion than boys.

White matter surface area increased by 1.7% (p<0.001) between ages 9 and 12. Surface area expansion was larger in the left hemisphere than in the right hemisphere (p = 0.03). Relative white matter volume increase correlated with relative white matter surface area expansion (r_p_ = 0.89; p<0.001). At a lobar level, all lobar white matter surface areas, apart from the occipital surface area bilaterally, increased significantly (p’s<0.02, frontal left 1.7%, frontal right 1.2%, parietal left 2.2%, parietal right 2.1%, temporal left 2.1%, temporal right 1.1%, occipital left 0.7%, occipital 0.5%). Figure 4 displays the percentage change in white matter surface area per anatomical cortical region.

### Sex Differences

Boys had a larger white matter volume than girls at both age 9 and 12 (p’s<0.001). There were no differences in absolute or relative volumetric white matter increase between boys and girls (p’s>0.30). Sex also had no significant effect on local volumetric change, as was measured with deformation based morphometry.

There were no differences in global or lobular white matter surface expansion between boys and girls. Locally, girls had a larger expansion in the right inferior temporal area (p = 0.006) and right medial-orbito frontal area (p = 0.04) and right paracentral lobule (p = 0.003). Considering white matter microstructure, girls had a slightly higher mean fractional anisotropy in pure white matter than boys at age 9 (0.405 versus 0.397, p = 0.022), but this difference disappeared at age 12. There was no difference in absolute or relative mean fractional anisotropy in pure white matter increase between boys and girls (p’s>0.21). The relationship between fractional anisotropy increase and white matter volumetric increase did not change when correcting for sex, nor when considering boys and girls separately.

### Genetic Analyses

Genetic influences were found for white matter volume (86%/85%), white matter surface area (83%/85%) and microstructural measures (7–54%/15–49%) at baseline and follow-up (see Table 5). Relative change in white matter volume and surface area, and relative change in fractional anisotropy over the three-year interval were not significantly influenced by genes. Instead, the correlation between relative white matter change and relative pure white matter fractional anisotropy change was completely explained by environmental factors (r_e_ = –0.67; confidence interval (–0.80, –0.48)), influencing both white matter growth and fractional anisotropy changes.

**Table 5:** Heritability and unique environmental influences for white matter volume, mean fractional anisotropy in pure white matter, white matter surface area and fractional anisotropy in fiber bundles at ages 9 and 12.

	9 years		12 years	
	Heritability (%)	Unique environment(%)	Heritability (%)	Unique environment (%)
White matter volume	86 [78–92]	14 [8]–[22]	85 [73–92]	15 [8]–[27]
Mean pure white matter FA	24 [1]–[49]	76 [51–99]	33 [3]–[59]	67 [41–97]
White matter surface area	83 [71–89]	17 [11]–[29]	86 [74–92]	14 [8]–[26]
FA Arcuate L	54 [32–71]	46 [29–68]	42 [16–64]	58 [36–84]
FA Arcuate R	34 [9]–[58]	66 [42–91]	31 [6]–[58]	69 [42–94]
FA cingulum L	51 [24–71]	49 [29–76]	36 [9–61]	64 [39–91]
FA cingulum R	54 [28–71]	46 [29–72]	35 [13]–[58]	65 [42–87]
FA fornix L	18 [3]–[41]	82 [59–97]	29 [6]–[53]	71 [47–94]
FA fornix R	21 [5]–[40]	79 [60–95]	39 [13–62]	61 [38–87]
FA genu	28 [5]–[51]	72 [49–95]	38 [9–61]	62 [39–91]
FA ILF L	28 [7]–[50]	72 [50–93]	21 [2]–[47]	79 [53–98]
FA ILF R	37 [12–61]	63 [39–88]	49 [19–70]	51 [30–81]
FA IFO L	7 [0–27]	93 [73–100]	15 [1]–[38]	85 [62–99]
FA IFO R	22 [3]–[48]	78 [52–97]	35 [6–62]	65 [38–94]
FA splenium	42 [21–60]	58 [40–79]	42 [19–61]	58 [39–81]
FA uncinate L	22 [3]–[45]	78 [55–97]	44 [15–67]	56 [33–85]
FA uncinate R	27 [2]–[51]	73 [49–98]	37 [10–62]	63 [38–90]

Values in brackets are 95% confidence intervals. Common environmental influences did not play a role in explaining variation in fiber bundles (based on both the Akaike criterion and Chi-square differences): only the results from the model incorporating genetic and unique environmental influences are displayed here. FA =  fractional anisotropy, L = left, R = right, ILF = inferior longitudinal fasciculus, IFO = inferior fronto-occipital fasciculus.

### Post-hoc Analysis: Histogram Comparisons

**Figure 5:**
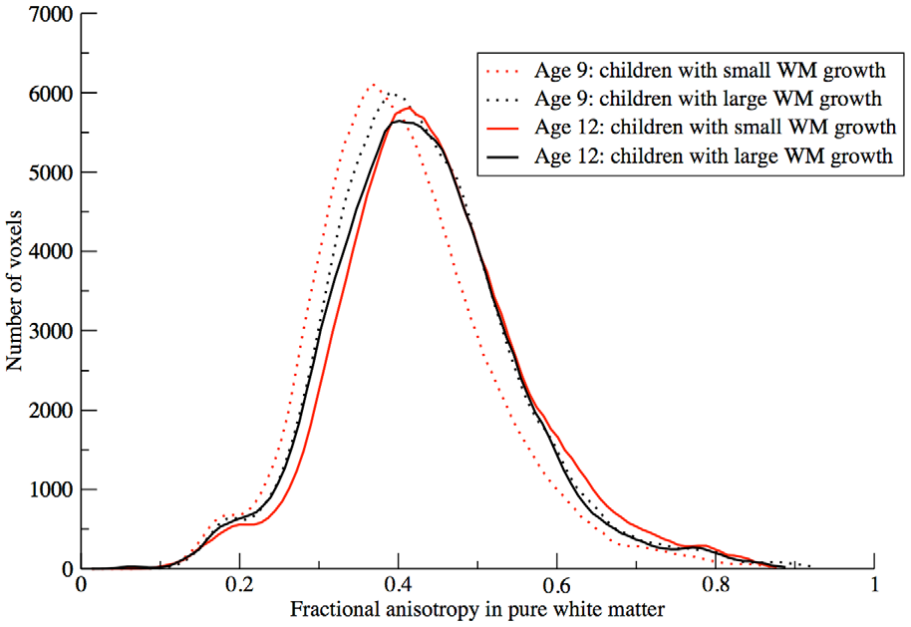
Histograms of fractional anisotropy in pure white matter at age 9 and age 12. In the 14 children with relatively large volumetric white matter growth mean fractional anisotropy was 0.43 at both ages. In 13 children with relatively small volumetric white matter growth, mean fractional anisotropy increased from 0.41 to 0.44.

Figure 5 shows histograms at age 9 and age 12 for children with relatively large, and relatively small white matter growth. As can be seen from this Figure, in neither group, the amount of voxels with low fractional anisotropy (possibly representing regions of crossing fibers) substantially differs between ages. A difference in mean white matter fractional anisotropy arises from a shift in the histogram to the right. This shift is larger in the group of children with relatively small volumetric white matter increase.

## Discussion

In this study we explored the local and global changes in white matter of the brain and how these changes are associated in children between 9 and 12 years of age. We find that white matter volume increases (on average 6.0%), white matter surface area expands (on average 1.7%), and thickening and lengthening of fiber bundles occurs, during this three-year period. All fiber bundles showed an increase in their fractional anisotropy with increasing age (1.3% to 6.9%). Genes influence white matter volume (>80%), white matter surface (>80%) and fractional anisotropy in fiber bundles (up to 54%). The developmental *changes* in white matter volume and microstructure were explained by some (unknown) unique environmental factor.

We found white matter surface area expansion in the frontal, parietal and temporal lobes, between 9 and 12 years of age. Our findings at two specific ages helps to localize, in both space and time, the increase in white matter volume [1] , mean surface area [39] and expansion of fiber bundles [40]–[41] that have been shown to occur in childhood and adolescence. Simultanously, fractional anisotropy increased in all the 14 major fiber bundles that we studied. Different rates of maturation have been suggested for different fiber bundles (e.g. [15] [42]–[43]) Our narrow age range makes it possible to pinpoint the changes that occur in a very specific time window. However, it does not allow for testing developmental trajectories over longer age-periods. The increases in fractional anisotropy found in our study are overall more widespread than those that have been found in voxel-based longitudinal studies in late adolescence and young adulthood [13]–[14]. As these studies were done in somewhat older samples (age ranges [16]–[21] and [14]–[19], respectively), it is possible that white matter microstructure is developing at a rather global level in children at the brink of puberty and that only later, during puberty, differentiation in the developmental pattern occurs. However, the recent longitudinal tract-based study in subjects aged 5–32 years, showed an age-related increase in 10 of the major fiber bundles [15] which is in line with our findings. Thus, we conclude that widespread age-related increases in fractional anisotropy occur between 9 and 12–years of age, although the extent is more prominent in some fiber bundles than in others.

The left arcuate fasciculus was the most prominently expanding fiber bundle of the 14 cortico-cortical fiber bundles between 9 and 12 years of age. Since the arcuate fasciculus is a bundle that is involved in language processing and young children have great potential to acquire new languages, we may speculate that the strengthening of the arcuate fasciculus is related to a stabilization of language skills. Indeed, the ability to acquire new languages diminishes fast after the age of 10 [44]. In addition, in the present study, prominent expansion was found in the splenium of the corpus callosum. The splenium contains the tracts involved in visual-spatial processing, which may be expected to develop early in life. Nevertheless, other studies too show prominent expansion in late childhood and young adolescence [45] and increases in fractional anisotropy until early adolescence [9]. It may be that the expansion occurring in our sample is related to bimanual coordination: during adolescence, both bimanual task performance and fractional anisotropy in the splenium increases [46].

Most likely, the continued expansion of white matter in childhood represents an increase in connectivity and reorganization throughout the brain. Based on post-mortem studies [47]–[48] the increase in white matter volume is usually attributed to an increase in myelin (but see [49]), thus representing an improvement in the speed of connections between anatomically distant brain areas. Our results indicate that white matter growth and fractional anisotropy increase cannot both be solely linked to myelination. While fractional anisotropy correlates with the thickness of myelin sheet [50]–[51], other factors such as compactness and organization of fiber bundles, and axonal diameter and density play a more prominent role (e.g. [52]–[55]). In the mouse-model of the shiverer mouse that lacks myelin, fractional anisotropy is less affected by dysmyelination than other diffusion parameters, such as the trace [56]. Although we can only speculate as to the underlying physiological processes underlying the development of white matter volume and microstructure, we hypothesize that the thickening of fiber bundles is not only the result from an increase of myelination, but also from an increase of axonal diameter and/or changes in the extracellular space. The latter two may negatively influence fractional anisotropy and their effects may temper the possible increase in fractional anisotropy due to increased myelination. Indeed, in pubertal rats, fractional anisotropy is positively correlated with the area of myelin sheath, but negatively with the extracellular space [51]. Thus, the negative correlation between white matter growth and fractional anisotropy increase indicates that (at least) two different biological processes occur during white matter development. Myelination is likely one of them, but other processes influencing coherence of axonal bundles must also play a role. Future studies are warranted to investigate the relation between changes in white matter volume/surface area and fractional anisotropy at a local level.

White matter volume in 8 and 9 year old children is highly heritable [18] [57]. We show here for the first time that white matter surface area is also strongly influenced by genes. There are only two studies investigating genetic influences in white matter microstructure during puberty and adolescence: our previous study in the same cohort of 9-year olds showed moderate genetic influences in several of the major fiber bundles [19]. A recent cross-sectional study comparing adolescents (12 and 16 year olds) to adults showed larger influences of genetic factors on fractional anisotropy in the younger group, indicating that heritability of white matter microstructure decreases with age, or environment increases its influence during life [58]. In our longitudinal study we did not find this decrease in heritability, possibly due to our younger age, and much shorter time interval. The developmental changes in white matter volume and microstructure were explained by some (unknown) unique environmental factor. Whereas unique environmental factors always include measurement error, the existence of an environmental correlation (and the fact that both measurements were obtained from a different scan) shows that there must be some external factor influencing both white matter growth and increase in anisotropy of fiber bundles. Considering the growing importance of the unique environment on children once they reach puberty, the white matter changes may reflect adaptation of the brain at this age.

## Supporting Information

Figure S1 **Figure S1. Significant tissue contraction: Significant Jacobian values <1 overlaid on the model brain (axial and coronal slice).** Values range from 0.98 (purple) to 0.70 (red). Contraction occurs around the ventricles and in gray matter at the gray/white matter boundary. We interpret this as representing enlargement of ventricles and cortical thinning, both of which have been shown in this sample [5] [36]. For visualization purposes, values were resampled to model brain resolution.
(TIFF)
