# Supplementary figures and images for: White Matter Development in Early Puberty: A Longitudinal Volumetric and Diffusion Tensor Imaging Twin Study
Source: PLoS One. 2012 Apr 13;7(4):e32316. doi: 10.1371/journal.pone.0032316 (PMC3326005; doi:10.1371/journal.pone.0032316)

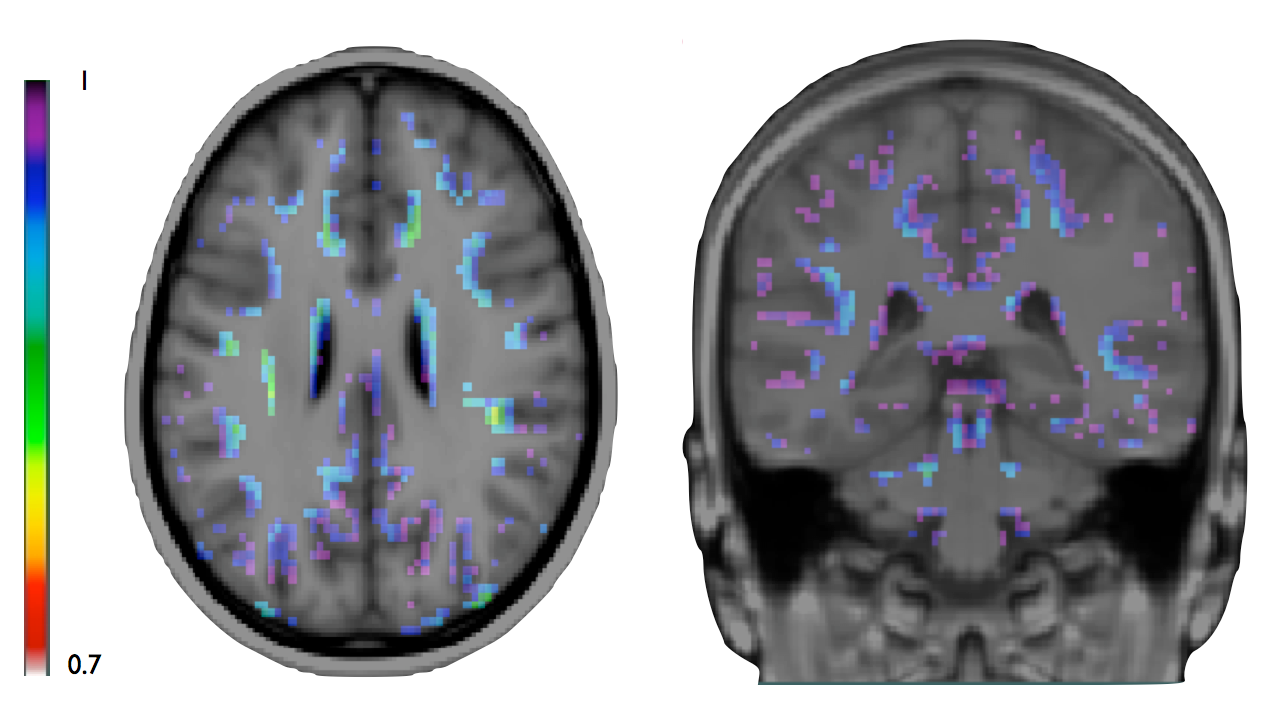

Supplement: Figure S1 — Significant tissue contraction: Significant Jacobian values <1 overlaid on the model brain (axial and coronal slice). Values range from 0.98 (purple) to 0.70 (red). Contraction occurs around the ventricles and in gray matter at the gray/white matter boundary. We interpret this as representing enlargement of ventricles and cortical thinning, both of which have been shown in this sample [5] [36]. For visualization purposes, values were resampled to model brain resolution. (TIFF) [file pone.0032316.s001.tif]
